# Supplementary material for: From sequence to enzyme mechanism using multi-label machine learning
Source: BMC Bioinformatics. 2014 May 19;15:150. doi: 10.1186/1471-2105-15-150 (PMC4229970; doi:10.1186/1471-2105-15-150)
Supplement: Additional file 2 — Java code of ml2db. Additional file ml2db_code.tar.gz contains the Java source code to run the multi-label machine learning experiments and save the results to database. The code’s Javadoc is included. [file 1471-2105-15-150-S2.zip › additional file 2/ml2db/ml2db/doc/mulan/data/LabelClustering.html]

LabelClustering


JavaScript is disabled on your browser.


- Overview
- Package
- Class
- Use
- Tree
- Deprecated
- Index
- Help

- Prev Class
- Next Class

- Frames
- No Frames

- All Classes

- Summary:
- Nested |
- Field |
- Constr |
- Method

- Detail:
- Field |
- Constr |
- Method


mulan.data

## Interface LabelClustering

- All Known Implementing Classes:
  :   GreedyLabelClustering

  ---

    

  ```
  public interface LabelClustering
  ```

  An interface for various label clustering algorithms.

  Version:
  :   05.05.2011

  Author:
  :   Lena Chekina (lenat@bgu.ac.il)

- - ### Method Summary

    Methods

    | Modifier and Type | Method and Description |
    | --- | --- |
    | `int[][]` | `determineClusters(MultiLabelInstances trainingSet)` Returns a label set partitioning into clusters |

- - ### Method Detail


    - #### determineClusters

      ```
      int[][] determineClusters(MultiLabelInstances trainingSet)
      ```

      Returns a label set partitioning into clusters

      Parameters:
      :   `trainingSet` - a set of training examples

      Returns:
      :   a label set partitioning


- Overview
- Package
- Class
- Use
- Tree
- Deprecated
- Index
- Help

- Prev Class
- Next Class

- Frames
- No Frames

- All Classes

- Summary:
- Nested |
- Field |
- Constr |
- Method

- Detail:
- Field |
- Constr |
- Method
